# Reconstructed human keloid models show heterogeneity within keloid scars

**DOI:** 10.1007/s00403-018-1873-1

**Published:** 2018-10-28

**Authors:** Grace C. Limandjaja, Leonarda J. van den Broek, Taco Waaijman, Melanie Breetveld, Stan Monstrey, Rik J. Scheper, Frank B. Niessen, Susan Gibbs

**Affiliations:** 1 Department of Molecular Cell Biology and Immunology, O|2 Lab Building, Amsterdam UMC, Vrije Universiteit Amsterdam, Amsterdam, Netherlands; 2 Department of Plastic Surgery, University of Ghent, Ghent, Belgium; 3 Department of Pathology, Amsterdam UMC, Vrije Universiteit Amsterdam, Amsterdam, Netherlands; 4 Department of Plastic Surgery, Amsterdam UMC, Vrije Universiteit Amsterdam, Amsterdam, Netherlands; 5 Department of Oral Cell Biology, Academic Centre for Dentistry Amsterdam (ACTA), University of Amsterdam and Vrije Universiteit Amsterdam, Amsterdam, Netherlands

**Keywords:** In vitro, Keloid, Keloid periphery, Keloid center, Cytokine, Extracellular matrix

## Abstract

Keloid scars are often described as having an actively growing peripheral margin with a regressing centre. The aim of this study was to examine the possible heterogeneity within keloids and the involvement of different regions within and around keloid scars in the pathogenesis, using an in vitro keloid scar model. In vitro skin models were constructed from keratinocytes and fibroblasts from normal skin and different regions within and around keloid scars: periphery, centre, and (adjacent) surrounding-normal-skin regions. Additionally, fibroblasts were isolated from the superficial-central and deep-central regions of the keloid and combined with central keratinocytes. All keloid regions showed increased contraction compared to normal skin models, particularly in central regions. Myofibroblasts were present in all keloid regions but were more abundant in models containing central-deep keloid fibroblasts. Secretion of anti-fibrotic HGF and extracellular matrix collagen IV gene expression was reduced in the central deep keloid compared to normal skin. No significant differences between peripheral and central regions within keloids were observed for inflammatory cytokine CCL20, CCL27, CXCL8, IL-6 and IL-18 secretion. Parameters for surrounding-normal-skin showed similarities to both non-lesional normal skin and keloids. In conclusion, a simple but elegant method of culturing keloid-derived keratinocytes and fibroblasts in an organotypic 3D scar model was developed, for the dual purpose of studying the underlying pathology and ultimately testing new therapeutics. In this study, these tissue engineered scar models show that the central keloid region shows a more aggressive keloid scar phenotype than the periphery and that the surrounding-normal-skin also shares certain abnormalities characteristic for keloids.

**Electronic supplementary material:**

The online version of this article (10.1007/s00403-018-1873-1) contains supplementary material, which is available to authorized users.

## Introduction

Keloid formation is an unfortunate complication of wound healing in which raised scar tissue proliferates beyond the boundaries of the original lesion [22]. This type of excessive scar tissue develops as an abnormal wound healing response to cutaneous injury [10, 22, 23]. Research on its pathogenesis has yet to uncover the etiology behind keloid formation and consequently our understanding of the mechanisms responsible for keloid development is limited. This is clearly illustrated by the inability of current treatment methods to satisfactorily manage keloids [3, 22]. As keloids develop exclusively in humans [29, 35] and research on their pathogenesis cannot be conducted solely on an intact original specimen, the need for a life-like in vitro model is evident.

Recently, we have demonstrated that keratinocytes and fibroblasts derived from human scars can be used to construct a full thickness skin model in vitro which shows resemblance to the native scar [16]. The keloid scar model shared several abnormalities with the hypertrophic scar model, but more importantly, differences were identified between these two abnormal scar types (hypertrophic scar and keloid). For the construction of these scar models, the scar tissue was used in its entirety. However, clinical observations suggest that keloids are not simply homogenous outgrowths. The distinction most often employed is that between the periphery and the centre of a keloid. The peripheral margin of the keloid is often described as being elevated, more red in color, actively proliferating and invading the surrounding normal skin; while the central region is seen as less elevated, lighter toned and clinically regressive over time [9, 13, 20, 24]. Differences have also been reported between keloid derived fibroblasts from peripheral or central regions when cultured in vitro with respect to lipid membrane composition [19], expression of apoptosis and extracellular matrix (ECM) related gene [24], collagen production [27], growth characteristics [20, 28], cell cycle distribution & regulation [8, 28, 30] and apoptosis-related protein expression [13, 20]. While the majority of published studies support the notion of an active periphery and a more quiescent centre, the opposite has also been reported with the central region thought to be the driving force behind keloid formation [28, 30]. Regardless, both concepts suggest that heterogeneity probably exists within a keloid scar, a finding that should not be ignored by those studying the mechanisms responsible for keloid formation.

As keloids are defined by their invasive growth into adjacent normal skin, it seems likely that the normal skin directly adjacent to keloids may in fact not truly be ‘normal’ and could, therefore, also play a role in keloid pathogenesis. Increased erythema in the normal skin directly adjacent to the keloid scars is often observed, and in a perfusion imaging study, blood flow in keloids and adjacent skin was indeed significantly higher than in nonadjacent normal skin [18]. Itching has also been reported to extend to peri-keloidal normal-appearing skin [14]. Taken together, these clinical observations suggest the skin directly adjacent to keloid scars may also be involved in keloid scar formation.

Given the aforementioned differences within keloid scars and the possible involvement of surrounding skin, we suspect that these different regions may differentially contribute to keloid scar formation. However, to our knowledge this has not yet been studied in a human, in vitro 3D scar model. In this study, we present an in vitro keloid scar model in which the different regions within and around the keloid scar can be studied and compared to unaffected normal skin to gain insight into keloid scar formation. The models serve a dual purpose of studying the underlying pathology and ultimately testing new therapeutics. To this end, the constructed in vitro models were compared to non-lesional skin with respect to the following scar parameters: contraction, epidermal and dermal thickness, expression of epidermal and dermal cell markers (Ki67, keratin 10, involucrin, vimentin, α-SMA), ECM gene expression and wound healing mediator secretion.

## Materials and methods

Normal skin (Nskin) was obtained from patients undergoing body contouring surgery to remove excess skin. Keloid scars (Kscar) were obtained from patients undergoing scar removal via excision and were selected by an experienced scar expert (plastic surgeon, author FBN). All scars used were at least 1 year old and had matured (with exception of one keloid donor: 6 months old). See Table 1 for donor characteristics.

**Table 1:** Tissue and donor characteristics

Donor	Tissue	Location	Etiology	Age	Previous treatment	Skin color	Pt age	Gender
1	Nskin	Breast	NA	NA	NA	−	40 yrs	Male
2	Nskin	Breast	NA	NA	NA	White	34 yrs	Male
3	Nskin	Abdomen	NA	NA	NA	Dark brown	−	Female
4	Nskin	Abdomen	NA	NA	NA	White	59 yrs	Female
5	Nskin	Abdomen	NA	NA	NA	−	49 yrs	Female
6	Nskin	Breast	NA	NA	NA	Dark brown	20 yrs	Female
7	Nskin	Leg	NA	NA	NA	White	−	−
8	Nskin	Abdomen	NA	NA	NA	Dark brown	39 yrs	Female
*Kscar is subdivided into P-Kscar, Cs-Kscar and Cd-Kscar*								
9	Kscar	Earlobe	Piercing	4 yrs	None	Light brown	46 yrs	Female
10	Kscar	Neck	Insect bite	8 yrs	Excision; Corticosteroids	Dark brown	15 yrs	Female
11	Kscar	Abdomen	Surgery	6 mo	None	Dark brown	23 yrs	Female
12	Kscar	Earlobe	Piercing	1 yr	Excision; Corticosteroids	Dark brown	17 yrs	Female
13	Kscar	Sternum	Skin irritation	12 yrs	Excision	Dark brown	39 yrs	Female
14	Kscar	Retroauricular	−	−	None	White	15 yrs	Male
15	Kscar	Pubic region	Inflammation	> 1 yr	None	Dark brown	46 yrs	Female
16	Kscar	Ear	Surgery	4 yrs	None	White	11 yrs	Female
*sNskin was derived from normal skin directly adjacent to keloid scars*								
17	sNskin	Pubic region	−	6 yrs	−	Dark brown	19 yrs	Female
13	sNskin	Sternum	Skin irritation	12 yrs	Excision	Dark brown	39 yrs	Female
8	sNskin	Abdomen	−	−	−	Dark brown	39 yrs	Female
18	sNskin	Breast	Surgery	> 1 yr	None	Dark brown	43 yrs	Female
15	sNskin	Pubic region	Inflammation	> 1 yr	None	Dark brown	46 yrs	Female

Overview of the characteristics of the tissue used for this study. All the skin models were constructed using donor matched keratinocytes and fibroblasts, note that all the tissues with the same donor number originate from the same patient

*NA* not applicable, *yr(s)* year(s), *mo* months, *Nskin* normal skin (*n* = 8), *P-Kscar* peripheral keloid (*n* = 8), *Cs-Kscar* central superficial keloid (*n* = 7), *Cd-Kscar* central deep keloid scar (*n* = 7), *sNskin* surrounding normal skin (*n* = 5), − no information available. See Table 3 for further clarification of skin model donor matching and number of biological replicates (‘*n*’) per experiment

### Cell culture and construction of skin models

**Fig. 1:**
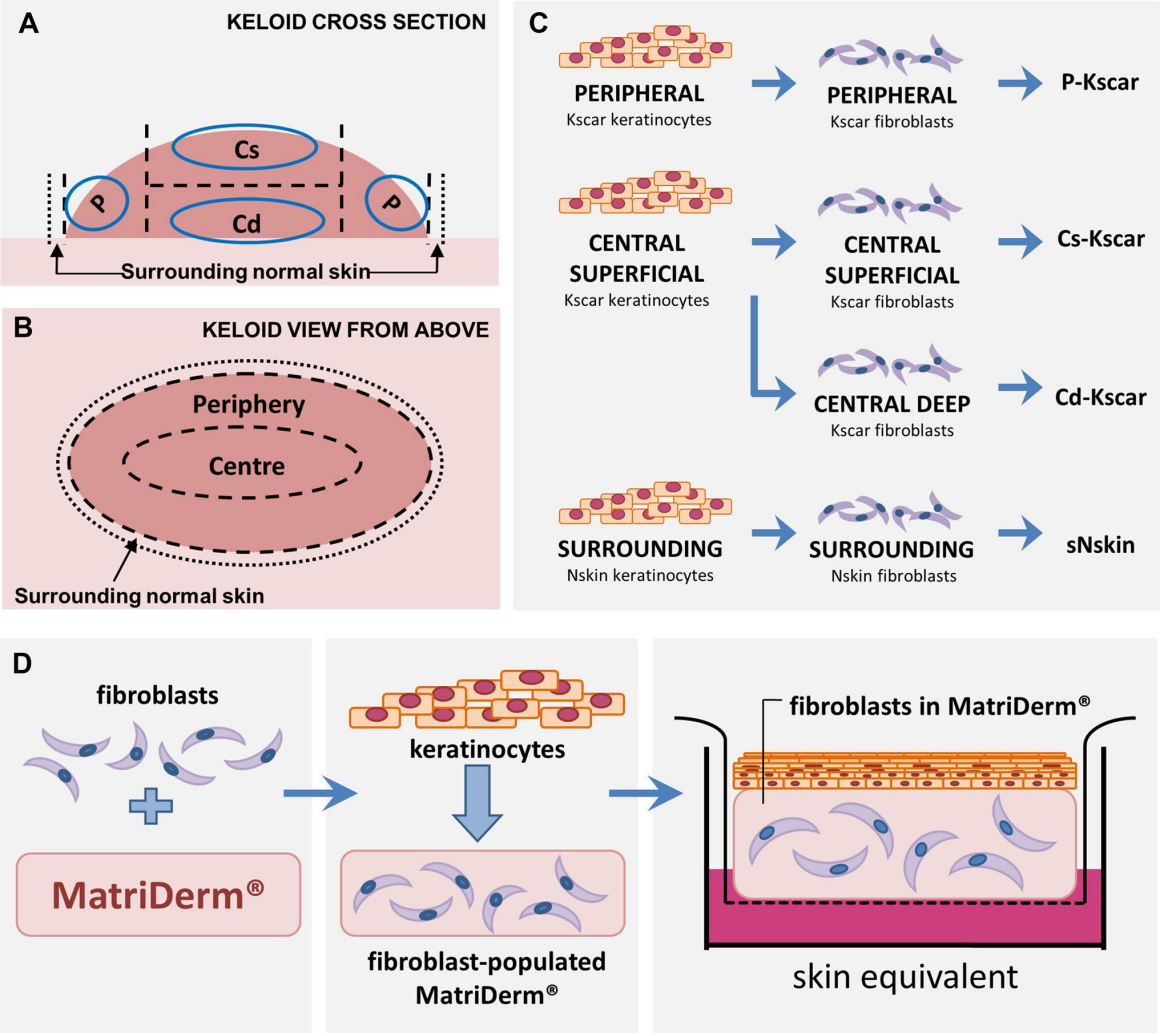
Construction of skin models. **a, b** Keloids are dissected into: peripheral region (P), the central superficial (Cs) and central deep regions (Cd); the normal skin directly adjacent to the keloid periphery (sN: surrounding normal skin). Keratinocytes and fibroblasts are isolated from each region (circles indicate where biopsies were taken for cell isolation) and combined to form a peripheral keloid (P-Kscar) model, central superficial keloid (Cs-Kscar) model, central deep keloid (Cd-Kscar) model, surrounding normal skin (sNskin) model (**c**). Skin models are constructed by first seeding fibroblasts into Matriderm® (**d**). After 3 weeks, keratinocytes are added and the skin models are then cultured air-exposed for an additional 2 weeks

After removal of subcutaneous fat and any other soft tissue until the typical firm and rubbery keloid consistency was reached, keloids were further subdivided into peripheral (P-Kscar), central superficial (Cs-Kscar) and central deep (Cd-Kscar) regions (Fig. 1a–c). Dermal tissue until ± 0.5 cm depth was included for cell isolation, Cs-Kscar and Cd-Kscar samples were obtained from the upper and lower central half, respectively. If present, any extralesional normal skin directly adjacent to the keloid (sNskin) was also included. sNskin extended to approximately ± 0.5 cm beyond P-Kscar (see area between edge of keloid and dotted line in Fig. 1a, b). In contrast to sNskin which was always derived from keloid patients, normal skin (Nskin) was obtained from unaffected control subjects. Keratinocytes and fibroblasts were isolated and cultured essentially as described previously [32, 34]. Skin models were constructed in duplicate from keratinocytes (P2) and fibroblasts (P2-3) essentially as previously described [32] ^21^ (Fig. 1d). In brief, 4 × 10^5^ Fibroblasts were seeded onto 2.2 × 2.2 cm squares of MatriDerm® (dr. Suwelack Skin & Health Care, Billerbeck, Germany) with FSM-I and cultured submerged in FSM-II for 3 weeks in 0.4 µm pore size transwells (Costar Corning Inc., New York, NY, USA) in a 37 °C, 5% CO_2_ atmosphere. Keratinocytes (P2) were then seeded on top of the fibroblast-populated MatriDerm® and cultured submerged in KC-I for 3–4 days, prior to culturing at an air–liquid interface in deep-well plates (BD Biosciences, Bedford, MA, USA) in KC-II for an additional 10 days. Upon addition of keratinocytes, SE were cultured in a 37 °C, 7.5% CO_2_ atmosphere. Medium was changed twice a week. See supplementary table 1 for contents of culture media (KC-I, KC-II, FSM-I, and FSM-II) used.

### Wound contraction

Wound contraction is expressed as a reduction in surface area of the skin models at the end of the culture period. Surface area (mm^2^) was determined in photographs of skin models at the time of harvesting (5 weeks culture), using NIS-Elements AR 2.10 imaging software.

### Histological analysis

Paraffin tissue sections (5 µm) were stained with Haematoxylin & Eosin (HE) for histologic evaluation and determination of epidermal and dermal thickness. *Epidermal thickness* quantified by counting the number of keratinocyte cell layers at three random points in each skin model section (200× magnification). *Dermal thickness* measured using NIS-elements software to calculate length in µm at five random points per skin model section (100× magnification).

### Immunohistochemical staining

Immunohistochemical stains were performed on deparaffinized, formalin-fixed tissue sections to assess epidermal proliferation (Ki67: clone MIB-1, Dakocytomation, Glostrup, Denmark; 1:50), epidermal differentiation (K10: keratin 10, clone DE-K10, Progen, Heidelberg, Germany; 1:500 and involucrin: clone SY5, Novocastra, New Castle, United Kingdom; 1:1000), presence of fibroblasts (vimentin: clone V9, Dakocytomation) and myofibroblasts (α-SMA: clone 1A4, Dakocytomation). Supplementary antigen retrieval treatments were performed prior to incubation with the primary antibody using a 15 min. incubation step with pepsin (K10) and/or heat-induced antigen retrieval with 0.01M citrate buffer pH 6.0 (Ki67, K10, K17, vimentin). *Immunohistochemical staining scoring* (−) absence of staining; (+) normal staining pattern; (++) increased number of positively stained cells; (+++) strongly increased number of positively stained cells. *Ki67 Proliferation index* 100 basal cells were counted in three random locations in a tissue section (100× magnification), after which the number of positive cells along this length of the epidermis was counted. The proliferation index was defined as the percentage of Ki67 positive nuclei within these regions.

### Enzyme-linked immunosorbent assay (ELISA)

Previously, we have identified a panel of wound healing mediators that are secreted predominantly by the epidermis (IL-1α, TNF-α, CCL5, VEGF), the dermis (TIMP2, HGF), or those significantly increased in the full thickness skin equivalents (CCL2, CXCL1, CXCL8, IL-6, sST2) [25]. CCL27 is found in burn wound exudates and has been implicated in the increased secretion of many of the aforementioned proteins [31]. IL-18 was also included in this panel because it has previously been implicated in keloid formation [4] and is known to be expressed in reconstructed human skin models also [11].

Culture supernatants (1.5 ml KC-II without hydrocortisone) derived from the skin models were collected over a 24-h period at the end of the culture period (5 weeks) to measure the levels of IL-6 and CXCL8 (PeliKine Sanguin Reagents, Amsterdam, The Netherlands); CCL2, CCL5, CCL20, CCL27, CXCL1, HGF and VEGF (R&D System Inc., Minneapolis, MN, USA); and IL-18 (MBL International, Woburn, MA, USA) secreted by the SE, using enzyme-linked immunosorbent assays (ELISA).

### Quantitative polymerase chain reaction (qPCR)

For RNA isolation, the epidermis was removed from the dermis using a slide-warmer (40 °C), the dermis was then flash frozen and stored in liquid nitrogen until further processing. Samples were disrupted and homogenized in a TissueLyser, then flash frozen for storage at − 80 °C. RNA isolation was performed using QiaShredder™ kits and RNeasy® Mini kits with on-column DNAse digestion and stored at − 80 °C. The Nanodrop spectrophotometer (Nanodrop technologies, Wilmington, DE, USA) was used to measure total RNA concentration. cDNA was synthesized using the RT^2^ First Strand Kit, while the RT^2^ SYBR Green Fluor qPCR Mastermix was used to run the real-time PCR reactions for the following genes (Table 2): *COL4A2, HAS1* and *MMP3*. These three genes were selected because they showed differential expression between Nskin and Kscar in previous work [16]. The geometric mean of two housekeeping genes (*ACTB* and *HPRT1*) was used to normalize expression. Unless stated otherwise, all RNA and qPCR reagents were obtained from Qiagen GmbH (Hilden, Germany).

**Table 2:** Overview of genes used for RT-PCR

Symbol	Description	Gene name	UniGene	RefSeq
*ACTB*	Actin, beta	BRWS1, PS1TP5BP1	Hs.520,640	NM_001101
*HPRT1*	Hypoxanthine phosphoribosyltransferase 1	HGPRT, HPRT	Hs.412,707	NM_000194
*COL4A2*	Collagen, type IV, alpha 2	ICH, POREN2	Hs.508,716	NM_001846
*MMP3*	Matrix metallopeptidase 3 (stromelysin 1, progelatinase)	CHDS6, MMP-3, SL-1, STMY, STMY1, STR1	Hs.375,129	NM_002422
*HAS1*	Hyaluronan synthase 1	HAS	Hs.57,697	NM_001523

An overview of the genes of interest (*COL4A2, HAS1, MMP3*) and the chosen housekeeping genes (*ACTB, HPRT1*). Listed here are gene symbols with full gene nomenclature and alternative synonymous gene names, matching UniGene (NCBI database of the transcriptome) cluster, and RefSeq (NCBI Reference Sequence project) number

### MTT assay

A colorimetric (MTT based) assay was used to quantify cell proliferation and viability (Roche Applied Science, Penzberg, Germany) of the skin models, as described previously [34].

### Data analysis

Experiments were performed in duplicate with *n* ≥ 4 different donors, except for some of the immunohistochemical staining (one of duplicate skin models was stained: keratin 10, vimentin, α-SMA) and qPCR experiments; see Tables 1 and 3 for an overview of donor matching and the number of biological replicates used per experiment. All results in graphs and tables were expressed as the mean ± standard error of the mean, PCR scatter plots showed the median. Normality testing (Shapiro–Wilk test) was performed on the residuals (errors); an ordinary one-way ANOVA with Tukey’s multiple comparisons tests was employed if the residuals passed the normality test (epidermal thickness; dermal thickness; contraction; MTT; ECM gene expression; secretion of VEGF, CCL5, CXCL8), otherwise the Kruskall-Wallis test with Dunn’s multiple comparisons tests (Ki67; α-SMA; secretion of CCL2, CCL20, IL-18, CXCL1, IL-6, HGF, CCL27) was applied. For analysis of the PCR data, gene expression (2^−∆Ct^) was normalized with the geometric mean of two housekeeping genes (*ACTB* and *HRPT1*). Differences were considered significant if *p* < 0.05 (*), *p* < 0.01(**) or *p* < 0.001 (***). It should also be noted that due to our use of stringent statistical analysis for these small sample sizes, our results very likely show underestimation of true statistical significance. A correction for performing multiple comparisons was made using an ANOVA. Additionally, the use of non-parametric tests in the event of non-normal distribution of the residuals, although statistically correct, further reduced power. For this reason, the term ‘trend’ was used when a clear pattern in graph data was observed without significance being reached; the exact *p* value was listed in the graph if 0.05 < *p* < 0.08. GraphPad Prism 6 software (GraphPad Software Inc., San Diego, CA, USA) was used to construct all graphs and tables and perform statistical analysis.

**Table 3:** Overview of the donors used per experiment

Nskin	sNskin	P-Kscar	Cs-Kscar	Cd-Kscar	RT-PCR	Additional experiments
d1						x
d2						x
d3						x
d4						x
d5					x	x
d6					x	x
d7					x	x
d8	d8				x	x
		d9	d9	d9		x
		d10	d10	d10		x
		d11	d11	d11	x	x
		d12	d12	d12	x	x
	d13	d13	d13	d13	x	x
		d14	d14	d14		x
	d15	d15			x	x
		d16	d16	d16	x	x
	d17					x
	d18				x	x

Overview of the donors used per experiment and donor-matching between tissue samples

*Nskin* normal skin, *P-Kscar* peripheral keloid, *Cs-Kscar* central superficial keloid, *Cd-Kscar* central deep keloid, *sNskin* surrounding normal skin, *x* entire row of donors was used for the experiments listed, *d* donor number, *RT-PCR* reverse transcription polymerase chain reaction. Additional experiments include: contraction, epidermal thickness, dermal thickness, immunohistochemical stainings and ELISA

## Results

### Increased epidermal thickness in keloid models

**Fig. 2:**
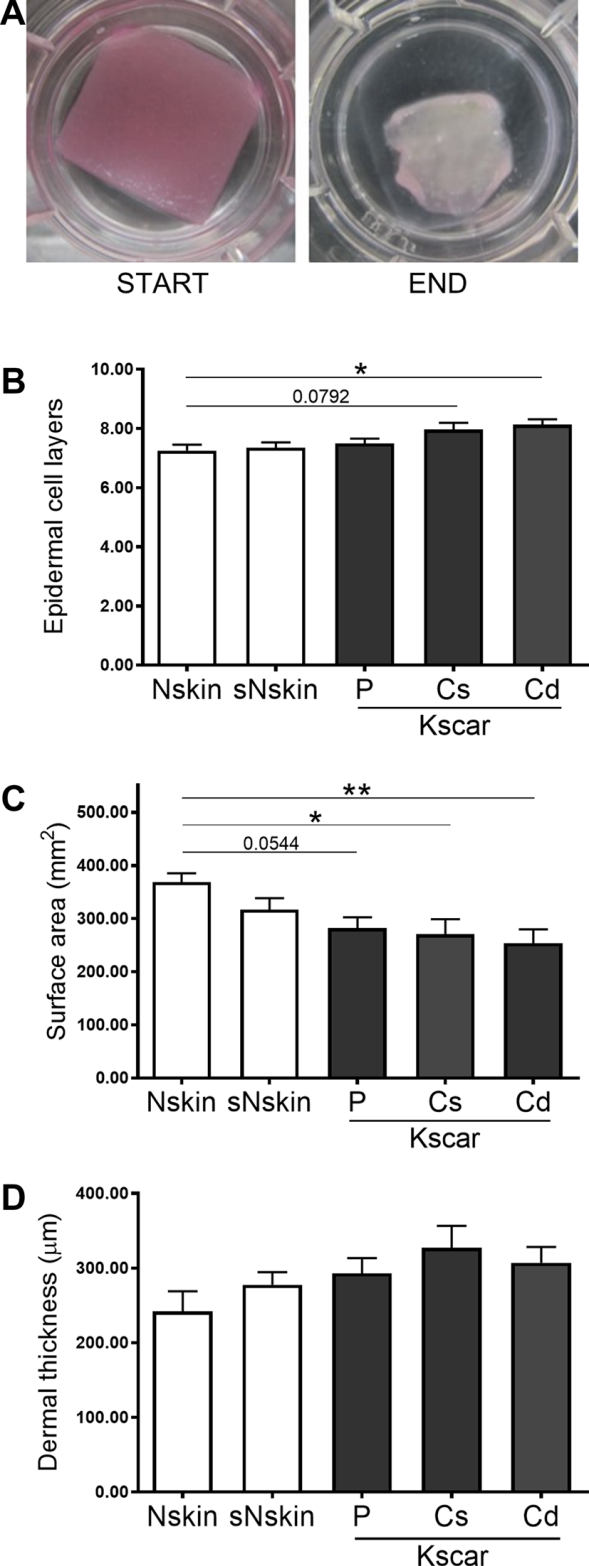
Increased contraction, epidermal and dermal thickness in central keloid regions. Absolute surface area of the skin models was measured in duplicate in *n* = 8 normal skin (Nskin), *n* = 8 peripheral keloid (P-Kscar), *n* = 7 central superficial keloid (Cs-Kscar), *n* = 7 central deep keloid (Cd-Kscar), *n* = 5 surrounding normal skin (sNskin). **a** shows macroscopic views of SE at the start and end of culturing; **b** shows the number of viable epidermal cell layers in the SE; **c** shows the dermal thickness measured in µm; **d** shows contraction measured as a reduction in end surface area after 5 weeks of culturing. *p < 0.05, if 0.05 > *p* < 0.08 then the exact *p* value is listed in the graph

Recently, we have shown that native keloids have increased epidermal thickness which was not related to hyperproliferation but may be related to abnormal involucrin expression [17]. Therefore, we first characterized the epidermal compartments of the keloid models (Fig. 2). All skin models showed a fully differentiated epidermis on a fibroblast-populated matrix. Cd-Kscar showed a significant increase in the number of epidermal keratinocyte layers compared to Nskin (Fig. 2b), with a similar trend occurring for Cs-Kscar (*p* = 0.0792). It should be noted that the differences described here were minor (1–2 cell layers) and not of the same magnitude as found in vivo (± 5 cell layers). sNskin showed similar results to unaffected Nskin.

**Fig. 3:**
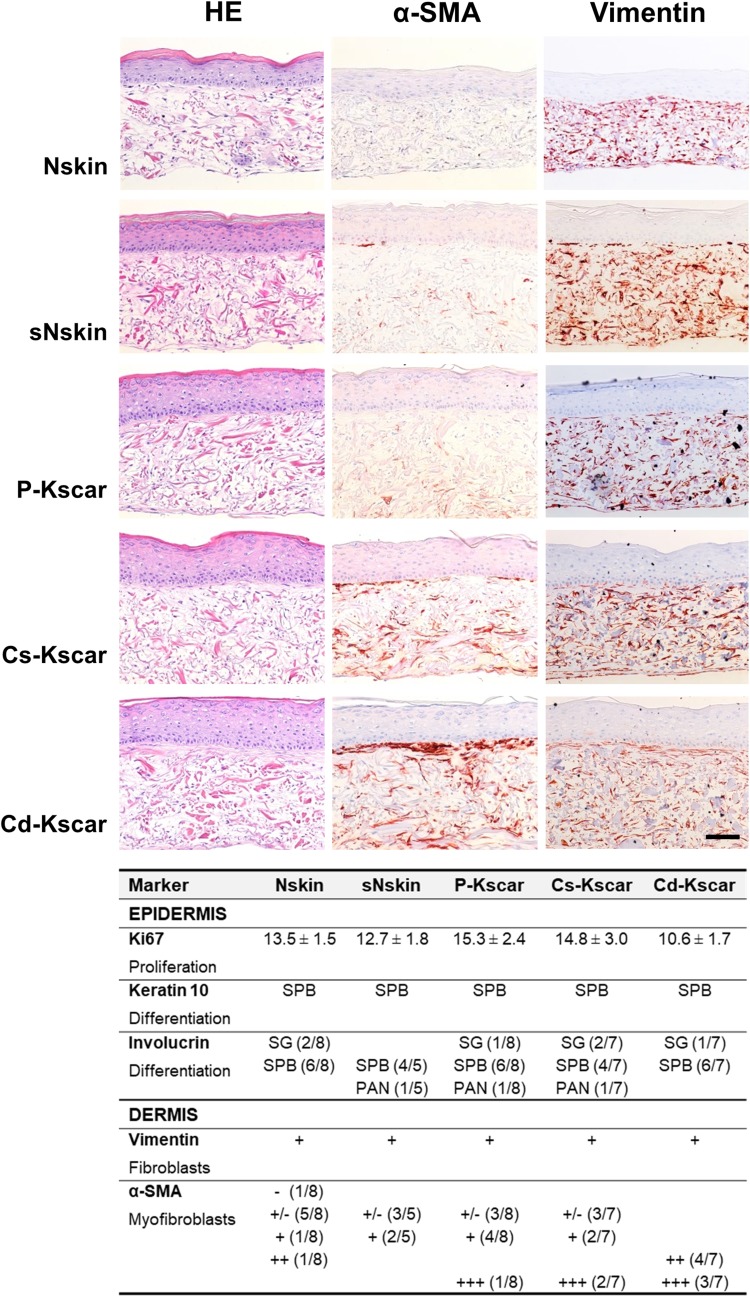
Increased α-SMA staining in central keloid regions. Upper panel shows representative pictures of HE, α-SMA and vimentin stainings performed on one of the duplicate skin models in *n* = 8 normal skin (Nskin), *n* = 8 peripheral keloid (P-Kscar), *n* = 7 central superficial keloid (Cs-Kscar), *n* = 7 central deep keloid (Cd-Kscar), *n* = 5 surrounding normal skin (sNskin). Magnification 200×, scale bar 100 µm. Lower panel shows the results of immunohistochemical stainings of epidermal markers (Ki67, keratin 10), dermal cellular markers (vimentin, α-SMA) in the skin models. Ki67 is expressed as mean ± SEM; SPB: suprabasal expression; SB: stratum basale; PAN: panepidermal (both SB and SPB); +/−: minimal expression; +: normal expression; ++: increased expression; +++: strongly increased expression; –: absent

In line with our previous findings on native tissue biopsies [17], there was no difference in the number of Ki67-positive proliferating basal cells between the different skin models (10–15% of basal cells) and normal suprabasal keratin 10 expression was observed in all the experimental groups (lower panel Fig. 3). We previously reported that while involucrin expression in healthy skin is confined to the stratum granulosum, in native Kscar involucrin is over expressed in all suprabasal layers [17]. In all the skin models described in this study, suprabasal involucrin was observed and was not just limited to the keloid constructs.

### Increased contraction and α-SMA expression in central keloid regions

Skin models constructed from all three different keloid scar regions showed a reduction in surface area and therefore increased contraction, compared to Nskin (Fig. 2a, c). sNskin was not significantly more contracted than Nskin. The increased contraction in Kscar was associated with the presence of myofibroblasts (Fig. 3). There was only little α-SMA staining in Nskin, sNskin and P-Kscar, but positive α-SMA staining was clearly present in Cs-Kscar and particularly Cd-Kscar. The difference in α-SMA expression was not the result of a disparity in the cellular contents of the dermis as there was no difference in vimentin staining between the models.

As increased thickness is one of the hallmarks of abnormal scars, we also assessed dermal thickness in the skin models. However, none of the keloid models nor sNskin showed significantly increased dermal thickness compared to Nskin (Fig. 2d).

### Keloid models show reduced dermal gene expression of collagen type IV α2

**Fig. 4:**
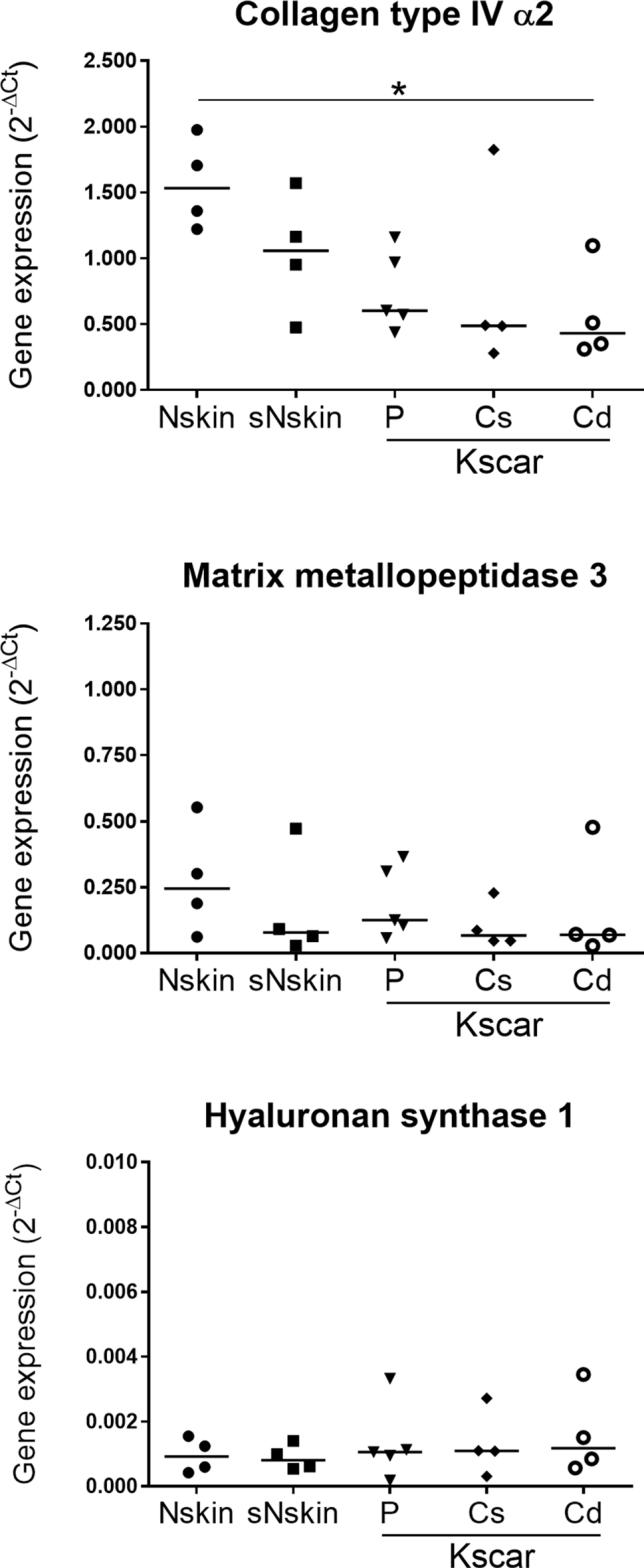
Differential dermal expression of ECM related genes. Dermal expression of *COL4A2, MMP3* and *HAS1* was determined in one of the duplicate skin models in *n* = 4 normal skin (Nskin), *n* = 5 peripheral keloid (P-Kscar), *n* = 4 central superficial keloid (Cs-Kscar), *n* = 4 central deep keloid (Cd-Kscar), *n* = 4 surrounding normal skin (sNskin). The scatter plots show the individual data points with the median, with *p < 0.05

Cd-Kscar showed significantly decreased dermal expression of collagen type IV α2 (*COL4A2*) compared to Nskin (Fig. 4). sNskin showed intermediate levels of *COL4A2* expression between Nskin and Kscar. In contrast to *COL4A2*, no difference in gene expression of other extracellular matrix genes, matrix metallopeptidase 3 and hyaluronan synthase 1, was observed between the skin models.

### Secretion profiles of wound healing mediators

**Fig. 5:**
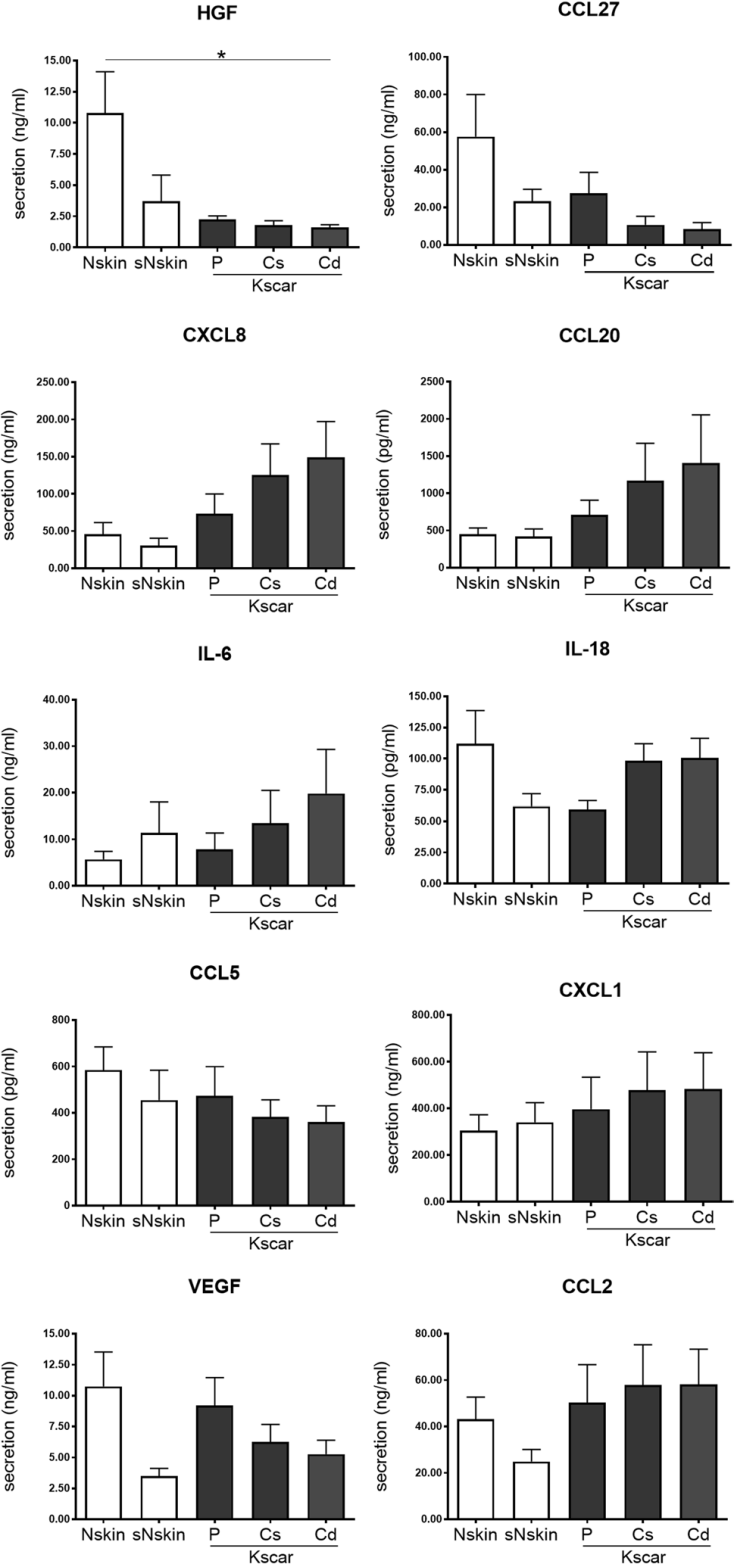
secretion of wound healing mediators. Wound healing mediator secretion of HGF, CCL27, CXCL8, CCL20, IL-6, IL-18, CCL5, CXCL1, VEGF, and CCL2 was determined in duplicate in *n* = 8 normal skin (Nskin), *n* = 8 peripheral keloid (P-Kscar), *n* = 7 central superficial keloid (Cs-Kscar), *n* = 7 central deep keloid (Cd-Kscar), *n* = 5 surrounding normal skin (sNskin). Graphs show mean ± SEM, with *p < 0.05

Next, we determined whether soluble wound healing mediators were differentially secreted by the keloid and normal skin models. HGF secretion was significantly decreased in Cd-Kscar compared to Nskin (Fig. 5). No significant differences between the different keloid models, sNskin and Nskin were observed for the other inflammatory cytokines: CCL20, CCL27, CXCL8, IL-6, IL-18, CXCL1, CCL2 and CCL5 (Fig. 5). Cytokine secretion levels were not influenced by differences in viability between the skin models, as MTT values were not significantly different between groups (data not shown).

## Discussion

In this study, we have used our previously published baseline keloid model [16] to further investigate the underlying keloid pathogenesis. We tested the hypothesis that differences exist within keloids which contribute differentially to keloid formation. Full thickness keloid scar models were constructed using keratinocytes and fibroblasts isolated from different regions within and around a keloid scar. Interestingly, differences were observed in scar phenotype between the different keloid regions: of these, the central deep keloid construct most often resembled the keloid phenotype, while the surrounding normal skin directly adjacent to the keloid showed a mixed normal skin and keloid scar phenotype.

Both the previously published baseline keloid model, as well as the central deep keloid model, showed increased contraction and α-SMA expression, decreased secretion of HGF, as well as decreased dermal expression of collagen type IV [16] compared to Nskin. Minor disparities between the two studies were found with regards to dermal *MMP3* and *HAS1* expression as well as epidermal thickness. The baseline keloid model comprising the entire keloid showed no increase in epidermal thickness, but reduced *COL4A2, HAS1* and *MMP3* gene expression in the dermal compartment of the keloid model compared to the normal skin model. In this study, we did observe an increase in epidermal thickness (albeit of a smaller magnitude than the native keloid tissue), but only collagen IV showed significantly reduced gene expression in the central deep keloid model (Cd-Kscar). We did not observe reduced *MMP3* or *HAS1* gene expression. While the baseline keloid model showed a trend towards increased dermal thickness (*p* = 0.075), there was no statistically significant difference between the keloid regions and normal skin in this study. Either way, in both the baseline keloid model as well as the current keloid regions models, increased epidermal and dermal thickness are not as excessively present in our *in vitro* models compared to the in vivo keloids [17]. This may be due to the relatively short culture period (5 weeks) and only two skin cell types currently being present in the models. However, herein now lies the value of our in vitro scar model, as it allows for the relatively easy addition of other cell types (e.g. immune cells and endothelial cells) in the future in a controlled and scalable manner.

Most studies on keloid heterogeneity report an active, proliferating and invasive role for the keloid periphery [1, 5, 12, 19, 24, 27, 33] compared to the quiescent centre. However, our results are in line with studies finding increased activity in the keloid central region [6, 21, 28]. To our knowledge, the only full thickness skin model constructed from keloid derived keratinocytes and fibroblasts from different regions within the keloid, was grafted into a mouse to develop a new keloid animal model [26]. Using superficial or deep keloid fibroblasts with keloid keratinocytes, two different keloid models were constructed and compared to normal skin model. After implantation into athymic mice, both keloid models were shown to be different from normal skin models (abnormal collagen organization) and differences were reported between superficial and deep keloid models. The deep keloid model had a thicker dermis and increased *COL1A1* expression, while the superficial keloid model only showed an increased wound area after grafting. Based on the method of cell isolation described, the deep keloid model very likely comprises cells of our central deep keloid construct (Cd-Kscar) and as such, the aforementioned findings correspond to our results. Nonetheless, a human full thickness skin model representing keloid heterogeneity is not currently available. In that regard, our in vitro keloid models described here could serve as an excellent starting point for further research.

A possible explanation for the dichotomy in findings reporting either the periphery or the central keloid region as the driving force behind continued keloid growth may be because different keloid phenotypes exist. Bella et al. [2] suggested that differences in genetic abnormalities may be responsible for heterogeneity between keloids, and distinguished between ‘superficial spreading’ keloids versus ‘raised’ keloids in an African tribe with familial keloids. In this regard, Supp et al. [26] also proposed an interesting model for the development of the ‘bulging’ keloid phenotype in which the deep keloid fibroblasts cause dermal thickening, while the superficial fibroblasts cause an increase in area by spreading the upper dermis (and overlying epidermis). The combination of deep dermal thickening and superficial dermal spreading then ultimately creates a ‘bulging’ keloid. Depending on the keloid phenotype, we propose that the actively growing region may be the periphery or the centre. This could explain why the periphery could very well be the actively expanding region in ‘spreading’ keloids, but not in the ‘bulging’ keloids where growth ensues from the deeper central regions. Retrospective analysis of the pictures of the keloid samples included in our study, in fact, showed that they were all of the ‘bulging’ phenotype. Three of the eight keloids used to construct the various keloid models were multinodular in appearance, consisting of several large dome-shaped nodules fused into a single large keloid. As the centre of each nodule was still more raised than the periphery, these were also considered ‘bulging’ keloids. Given that all our keloid donors were of the ‘bulging’ phenotype, this could explain why only the central deep region was statistically significantly different from normal skin.

In this study, we also included extra-lesional normal skin (sNskin) directly adjacent to but separate from P-Kscar. Even though this surrounding normal skin (sNskin) did not show any statistically significant differences with the other models for the individual parameters studied, when taking all parameters together a clear pattern was emerging. sNskin was usually intermediate between normal skin and keloid in the expression of scar parameters and often similar to the peripheral keloid model (contraction; α-SMA expression; secretion of HGF; expression of *COL4A2*). Abnormalities in the surrounding normal skin (sNskin) have been previously reported. Lee et al. [15] found that the into the surrounding normal skin. Using expression microarrays, Hahn et al. [7] found that increased expression of many of the genes in keloid-derived keratinocytes and fibroblasts corresponded with similarly increased expression in cells derived from adjacent non-lesional skin. However, we are unable to compare our results to the findings of others because it was often unclear what other authors considered to be surrounding normal skin (sNskin). Definitions of sNskin could also mean the normal skin in the same anatomical location but not necessarily in the direct vicinity of the keloid and alternatively, sometimes the surrounding normal skin was included with the peripheral margin generating what we would consider an sNskin/P-Kscar region.

Notably, from all the parameters studied, significant differences or trends were only obtained in a few parameters. This is indeed a limitation of the study and is most probably a result of the experimental set-up which could not be avoided. Surrounding normal skin was very rarely included with the keloid samples provided to us by the plastic surgeons, and when it was included, the keloid itself was often too small to enable adequate cell isolation from the different keloid regions. Therefore, non-donor paired samples were included in the analysis. Additionally, small sample sizes are inherent to the time-consuming nature of tissue engineering. The problem with statistical analysis of small sample sizes (*n* < 24) is that one either errs on the side of over- or underestimation, depending on whether a correction is applied for performing multiple comparisons. In this study, we had small sample sizes and corrected for multiple comparisons using a one-way ANOVA, power was further reduced in our study by our use of non-parametric testing when the residuals were not normally distributed. Thus, we have been relatively strict with our statistics to the point of underestimation, but consider this the better option as opposed to risking overestimation.

To conclude, we were able to generate different keloid scar models from keratinocytes and fibroblasts derived from intralesional peripheral, central superficial and central deep regions, as well as extralesional surrounding normal skin. Of these regions, only the central deep keloid regions showed statistically significant differences when compared to normal skin and thus displayed the most aberrant behavior. As all the keloid cells were derived from ‘bulging’ type keloids, this suggests that the central deep keloid region is likely the driving force behind the development of keloids of this ‘bulging’ phenotype. Our study has demonstrated the need for a clear and unambiguous description of the keloid type (e.g. ‘spreading’ or ‘bulging’) and of the exact location within keloid scars from which samples are taken. Additionally, we would encourage the inclusion of the skin adjacent to the keloids, as it is important to find out to what extent this region contributes to keloid scar formation and consequently if it should be targeted for treatment as well. This study is the first demonstration of how the in vitro baseline keloid scar model we have previously established [16] can be utilized not only as a future animal-free drug testing platform but also to further our understanding of the underlying pathogenesis.

## Electronic supplementary material

Below is the link to the electronic supplementary material.

Supplementary material 1 (DOCX 15 KB)

## Abbreviations

Nskin: Normal skin
Kscar: Keloid scar
P: Peripheral
Cs: Central superficial
Cd: Central deep
ECM: Extracellular matrix
LAMA1: Laminin alpha 1
COL4A2: Collagen type IV alpha 2
ITGA5: Integrin alpha 5
HAS1: Hyaluronan synthase 1
MMP1: Matrix metalloproteinase 1
MMP3: Matrix metalloproteinase 3
