# Supplementary material for: Reconstructed human keloid models show heterogeneity within keloid scars
Source: Arch Dermatol Res. 2018 Oct 28;310(10):815–26. doi: 10.1007/s00403-018-1873-1 (PMC6244653; doi:10.1007/s00403-018-1873-1)
Supplement: Supplementary file 1 — Supplementary material 1 (DOCX 15 KB) [file 403_2018_1873_MOESM1_ESM.docx]

**Supplementary material**

**-**

**RECONSTRUCTED HUMAN KELOID MODELS SHOW**

**HETEROGENEITY WITHIN KELOID SCARS**

Grace C. Limandjaja^1^, Leonarda J. van den Broek^1^, Melanie Breetveld^1^, Taco Waaijman^1^, Stan Monstrey^2^, Rik J. Scheper^3^, Frank B. Niessen^4^, Susan Gibbs*^1,5^

^1^ Department Molecular Cell Biology and Immunology, VU Medical Centre (VUMC), Amsterdam, the Netherlands

^2^ Department of Plastic Surgery, University of Ghent, Ghent, Belgium

^3^ Department of Pathology, VU Medical Centre (VUMC), Amsterdam, the Netherlands

^4^ Department of Plastic Surgery, VU Medical Centre (VUMC), Amsterdam, the Netherlands

^5^ Department of Oral Cell Biology, Academic Centre for Dentistry Amsterdam (ACTA), University of Amsterdam and Vrije Universiteit Amsterdam

* Correspondence to: Susan Gibbs, Department of Dermatology, O/2 building, room 11E05, VU University Medical Centre, Amsterdam, the Netherlands, e-mail: s.gibbs@vumc.nl

**Supplementary table 1**: culture media

| **Medium** | **Components** |
| --- | --- |
| KC-I  (keratinocyte medium type I) | DMEM (Lonza, Verviers, Belgium) : F12-HAM nutrient mixture + L-glut. (HAMF12; Gibco, Grand Island, NY, USA) in a 3:1 ratio with 1% UltroserG (Biosepra, Cergy-St-Christophe, France), 1% PenStrep (Gibco, Grand Island, USA), 2ng/ml human Keratinocyte Growth Factor, 0.09 µmol/L Insulin, 1µmol/L Hydrocortisone, 1µmol/L Isoproterenol hydrochloride |
| Fibroblast medium | DMEM (Lonza), 1% UltroserG (Biosepra), 1% PenStrep (Gibco) |
| FSM-I  (fibroblast sheet medium type I) | DMEM (Lonza), 2% UltroserG (Biosepra), 1% PenStrep (Gibco), 5µg/ml Insulin 10-3M, 50µg/ml L-Ascorbic acid and 5ng/ml Epidermal Growth Factor |
| FSM-II  (fibroblast sheet medium type II) | DMEM:HAMF12 (Lonza; Gibco) in a 3:1 ratio, 1% PenStrep (Gibco), 2% UltroserG (Biosepra), 5 µg/ml Insulin 10-3M, 50µg/ml L-Ascorbic acid and 5ng/ml Epidermal Growth Factor |
| KC-II  (keratinocyte medium type II) | DMEM:HAMF12 Lonza; Gibco) in a 3:1 ratio, 1% PenStrep (Gibco), 0.2% UltroserG (Biosepra), 0.1 µmol/L Insulin, 1µmol/L Hydrocortisone, 1µmol/L Isoproterenol hydrochloride, 10 µmol/L L-Carnitine hydrochloride, 0.01 µmol/L L-Serine, 1µmol/L DL-α-Tocopherol, 0.4mmol/L L-Ascorbic acid, supplemented with a lipid mixture containing 7 µmol/L Arachidonic acid, 25 µmol/L Palmitic acid, 15 µmol/L Linoleic acid and 24 µmol/L Bovine Serum Albumin |
| Monocyte medium | DMEM (Lonza), 10% heat inactivated FBS (HyClone®, Northumberland, UK), 1% PenStrep (Gibco) |
| Medium for collection of 24-hr supernatant | KC-II, but without Hydrocortisone |

**Supplementary Table 1**: shows an overview of all the different culture media used for the construction of the skin models. All reagents were obtained from Sigma-Aldrich (St. Louis, MO, USA) unless otherwise specified.
